# PET Evaluation of the Novel F-18 Labeled Reversible Radioligand [^18^F]GEH200449 for Detection of Monoamine Oxidase-B in the Non-Human Primate Brain

**DOI:** 10.1021/acschemneuro.3c00332

**Published:** 2023-08-17

**Authors:** Katarina Varnäs, Sangram Nag, Christer Halldin, Lars Farde

**Affiliations:** Karolinska Institutet, Department of Clinical Neuroscience, Center for Psychiatry Research and Stockholm County Council, BioClinicum J:15, Visionsgatan 4, SE-171 64 Solna, Sweden

**Keywords:** PET imaging, monoamine oxidase B, radioligand development, reversible radioligands, fluorine-18, [^18^F]GEH200449

## Abstract

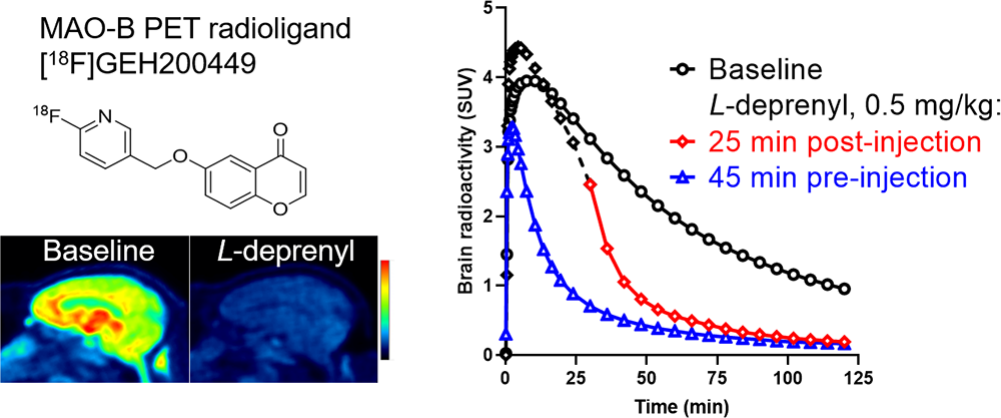

Positron emission tomography (PET) using radioligands for the enzyme monoamine oxidase B (MAO-B) is increasingly applied as a marker for astrogliosis in neurodegenerative disorders. In the present study, a novel reversible fluorine-18 labeled MAO-B compound, [^18^F]GEH200449, was evaluated as a PET radioligand in non-human primates. PET studies of [^18^F]GEH200449 at baseline showed brain exposure (maximum concentration: 3.4–5.2 SUV; *n* = 5) within the range of that for suitable central nervous system radioligands and a regional distribution consistent with the known localization of MAO-B. Based on the quantitative assessment of [^18^F]GEH200449 data using the metabolite-corrected arterial plasma concentration as input function, the Logan graphical analysis was selected as the preferred method of quantification. The binding of [^18^F]GEH200449, as calculated based on regional estimates of the total distribution volume, was markedly inhibited (occupancy >80%) by the administration of the selective MAO-B ligands *L*-deprenyl (0.5 and 1.0 mg/kg) or rasagiline (0.75 mg/kg) prior to radioligand injection. Radioligand binding was displaceable by the administration of *L*-deprenyl (0.5 mg/kg) at 25 min after radioligand injection, thus supporting reversible binding to MAO-B. These observations support that [^18^F]GEH200449 is a reversible MAO-B radioligand suitable for applied studies in humans.

## Introduction

Monoamine oxidases A and B (MAO-A, MAO-B) are enzymes involved in the metabolism of monoamine neurotransmitters. The two enzymes have since long been implicated in the pathophysiology and treatment of several neurological and psychiatric conditions.^1^ Non-selective monoamine oxidase inhibitors have antidepressant properties, and MAO-B selective inhibitors are widely used in the pharmacological treatment of Parkinson’s disease.^2^ Moreover, MAO-B is abundantly localized in brain glial cells^3,4^ and has been found to be overexpressed in reactive astrocytes.^3^ In Alzheimer’s disease, overexpression and increased levels of MAO-B have been linked to the accumulation of amyloid β-peptides responsible for the development of amyloid brain deposits.^5^

Imaging of MAO-B binding using positron emission tomography (PET) has been used to examine drug-induced effects on MAO-B^6^ and is increasingly applied as a potential surrogate biomarker for monitoring of astrogliosis in neurodegenerative diseases.^7,8^ PET imaging of MAO-B can be achieved using labeled enzyme inhibitors or metabolic trapping agents as radioligands.^7,9^ Several MAO-B PET radioligands have been developed, of which the irreversible enzyme inhibitor [^11^C]*L*-deprenyl-D2 has been the most widely applied.^7^ Potential limitations of [^11^C]*L*-deprenyl-D2, including irreversible binding to MAO-B and the formation of radioactive metabolites that may enter the brain, have motivated the developments of novel radioligands. One example is [^11^C]SL25.1188 that has been characterized as a reversible, selective MAO-B binding radioligand^10,11^ and has been employed in studies of patients with depression^12^ and post-traumatic stress disorder.^13^

In addition, MAO-B tracers have been labeled with the more long-lived isotope fluorine-18 (half-life 110 min vs 20.4 min for carbon-11) to allow for wider clinical research. In this respect, a fluorine-18 analogue of SL25.1188 has been developed and characterized as a MAO-B PET radioligand in non-human primates (NHPs).^14^ More recently, the MAO-B radioligand [^18^F]SMBT-1^15^ has reached clinical development and been applied in studies in Alzheimer’s disease patients.^16,17^

In collaboration with GE Healthcare, we have previously characterized the binding properties of five reversible fluorine-18 labeled MAO-B ligands using autoradiography.^18^ Of the compounds evaluated, [^18^F]GEH200449 showed promising characteristics for further evaluation as a PET radioligand, including specific binding to MAO-B and low non-specific binding in human brain tissue. In the present study, the suitability of [^18^F]GEH200449 as a PET radioligand was further evaluated in vivo in NHPs of the species *Macaca fascicularis* (*M. fascicularis*).

## Results and Discussion

Altogether 13 PET measurements were undertaken in five NHPs (Supporting Information Table S1). Due to technical challenges involved with arterial cannulation, it was not possible to obtain arterial blood data for the two PET measurements in NHP #2 and for the pretreatment studies with AZD9272, rasagiline (0.25 mg/kg), and fenobam in NHPs #3–5.

**Figure 1:**
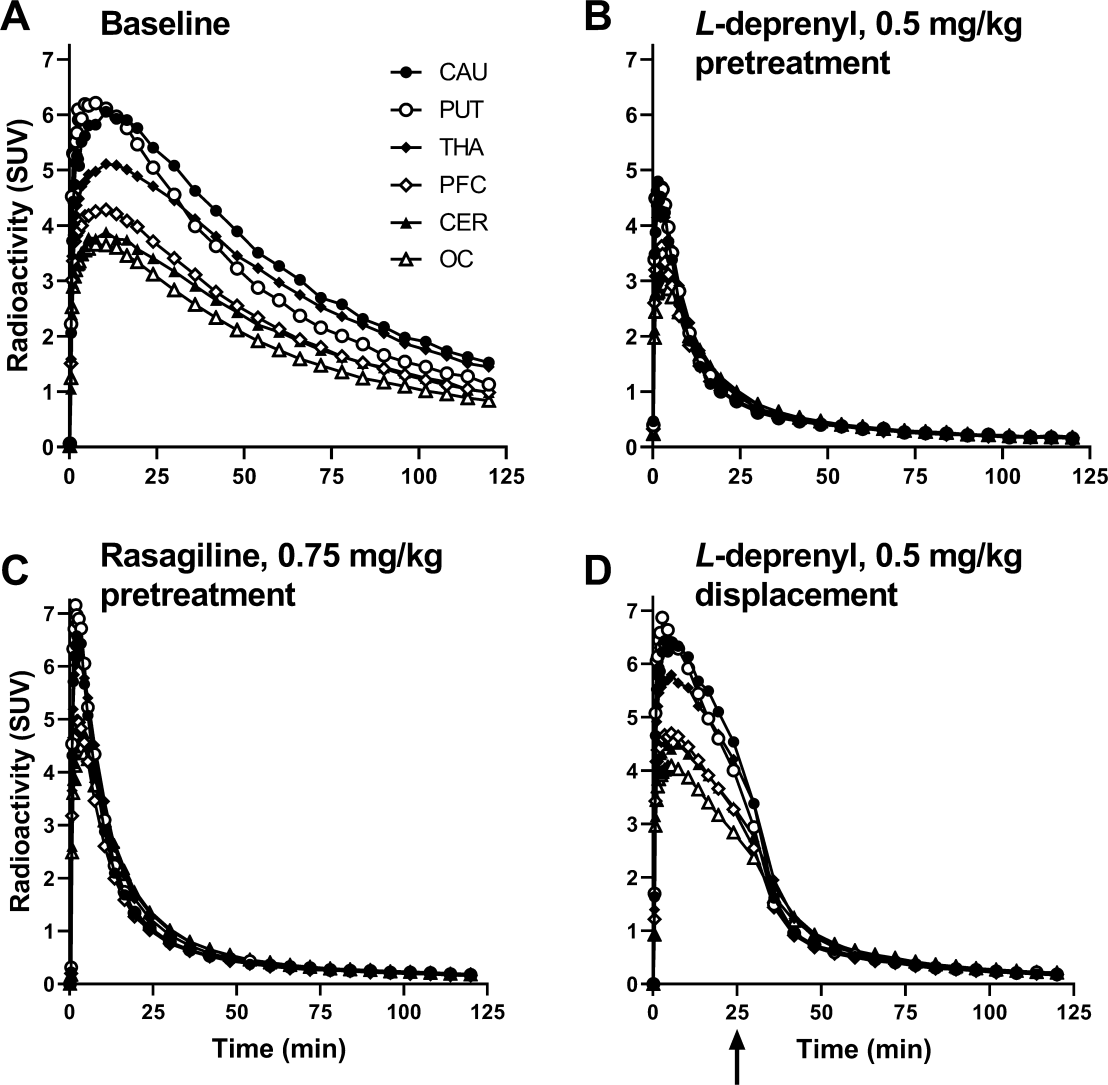
Time curves for [^18^F]GEH200449 brain regional radioactivity in NHP #4 at baseline (A) and after pretreatment with 0.5 mg/kg of *L*-deprenyl (B) or 0.75 mg/kg of rasagiline (C) at 45 min before radioligand injection and after displacement with 0.5 mg/kg of *L*-deprenyl at 25 min after radioligand injection (D). Arrow indicates start of *L*-deprenyl injection. CAU, caudate nucleus; PUT, putamen; THA, thalamus; PFC, prefrontal cortex; CER, cerebellum; OC, occipital cortex; and SUV, standardized uptake value.

**Figure 2:**
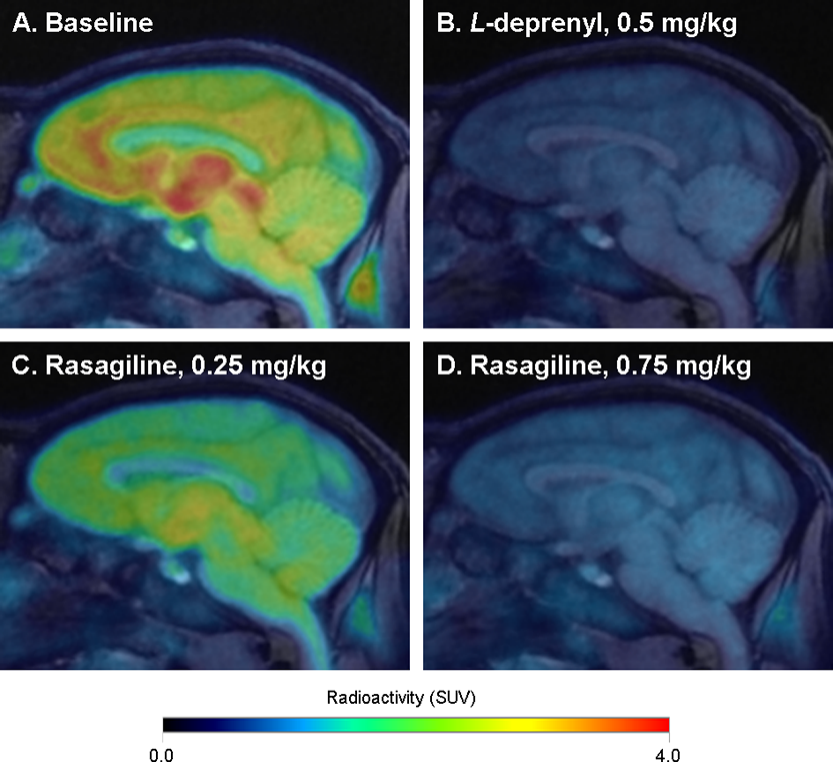
Fused MR and PET images showing brain radioactivity after injection of [^18^F]GEH200449 in NHP #4 at baseline (A) and following administration of 0.5 mg/kg *L*-deprenyl (B), 0.25 mg/kg rasagiline (C), or 0.75 mg/kg rasagiline (D). Average images for 123 min. SUV, standardized uptake value.

The time curves for whole-brain radioactivity at baseline rapidly reached a maximum concentration of 3.4–5.2 SUV (4.0–6.4 %ID) within 1.8–7.5 min after IV injection of [^18^F]GEH200449 (Supporting Information Figure S1). The high whole-brain radioactivity is suitable for detailed analysis of regional binding. The PET images and the time curves for regional radioactivity showed a pattern of high binding in the striatum and thalamus, whereas the binding was lower in the cortex and cerebellum (Figures 1 and 2A). This regional distribution is consistent with the localization and levels of MAO-B that have been demonstrated in vitro^19,20^ and supports the interpretation that [^18^F]GEH200449 binds specifically to MAO-B also in vivo.

Regional radioactivity was inhibited by pretreatment with the selective MAO-B ligands *L*-deprenyl or rasagiline administered 45 min before radioligand injection (Figure 1B,C; Figure 2B–D). In displacement experiments, [^18^F]GEH200449 binding was markedly reduced following administration of *L*-deprenyl 25 min after radioligand injection (Figure 1D). Two doses of rasagiline (0.25 and 0.75 mg/kg) were administrated in separate pretreatment experiments in NHP #4 (Figure 2C,D). The inhibitory effect was more pronounced after the higher dose indicating dose dependency. Taken together these observations with reference competitors support the view that [^18^F]GEH200449 binding in the NHP brain is reversible and selective toward MAO-B.

A reduction in the regional radioactivity for [^18^F]GEH200449 was also observed after the administration of AZD9272 (0.15 mg/kg) in NHP #3 and after the administration of fenobam (1.0 mg/kg) in NHP #5 (Figure S2). Fenobam and AZD9272 are metabotropic glutamate receptor 5 (mGluR5) compounds that recently have been found to have a secondary binding site at MAO-B.^21^ This reported finding has been based on in vitro and in vivo competition binding studies using radiolabeled AZD9272 and *L*-deprenyl.^21^ The present additional pretreatment studies using unlabeled fenobam and AZD9272 provide further support that [^18^F]GEH200449 binds to MAO-B.

**Figure 3:**
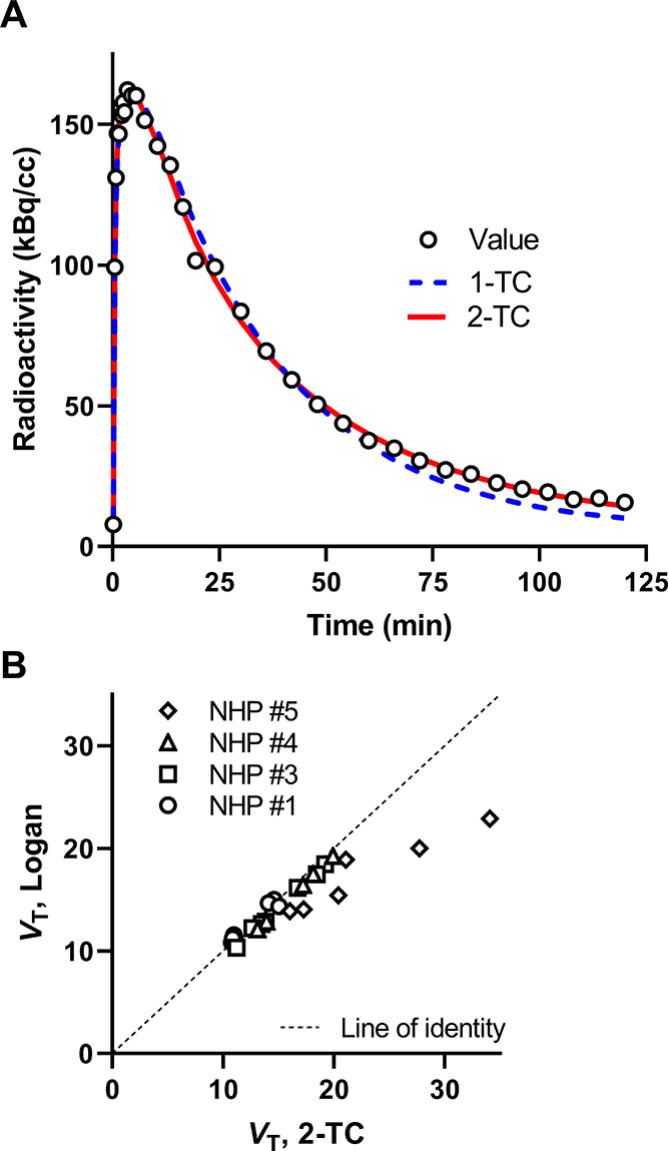
(A) Time curve for radioactivity in putamen with model fits for the one- (1-TC) and two-tissue compartment (2-TC) models for NHP #3. (B) Total distribution volume (*V*_T_) obtained by the Logan graphical analysis plotted versus that obtained by the 2-TC model. *V*_T_ was not included for the cerebellum in NHP #4, or for the occipital cortex in NHPs #4 and #5 since the 2-TC model fits yielded implausible estimates of *V*_T_ (>500 mL cm^–3^) for these measurements and regions.

After the administration of [^18^F]GEH200449, the fraction of parent radioligand in plasma declined rapidly. At 30 min after injection, 5–28% of parent radioligand remained unchanged (Figure S3). Time curves for regional radioactivity obtained in the four baseline measurements that included metabolite-corrected arterial blood sampling were interpreted using standard kinetic one- (1-TC) and two-tissue compartment (2-TC) models and by the Logan linear graphical analysis method. For most measurements and brain regions analyzed, the 2-TC model was statistically preferred over the 1-TC model (Figure 3A; Table S2). Logan’s graphical analysis yielded a linear phase from 40 min for all measurements and regions analyzed.

Estimates of the total distribution volume (*V*_T_) obtained by the 2-TC model correlated with those obtained by the Logan graphical analysis (Pearson’s *r* = 0.92, *P* < 0.0001; Figure 3B; Table 1). However, for two regions in NHP #4 and one region in NHP #5, the *V*_T_ values obtained by the 2-TC model showed large parameter standard errors and could not be estimated with good precision. For three additional regions in NHP #5 (caudate nucleus, thalamus, and prefrontal cortex), the *V*_T_ values obtained by the 2-TC model were overestimated by 30–50% relative to values obtained by the Logan method (Figure 3B). Uncertainty in the estimates of *V*_T_ was associated with *k*_4_ values approaching 0 for these regions (Table S2). The small *k*_4_ values are unlikely to represent irreversible binding to MAO-B, given that the binding is completely displaceable by administration of a MAO-B inhibitor after radioligand injection (Figure 1D). Alternatively, such results could reflect the presence of a slowly equilibrating compartment as would be expected for a radioactive metabolite that enters the brain and contributes to the signal. However, this explanation also seems unlikely, since the effect of metabolites is expected to be consistent across brain regions. Nevertheless, the possible contribution of radiometabolites to the brain signal for [^18^F]GEH200449 should be addressed in future studies. Given the uncertainty in parameter estimates obtained by the 2-TC method, the Logan graphical analysis should be the preferred method for quantitative analysis of [^18^F]GEH200449 binding.

**Table 1:** Regional Total Distribution Volume (*V*_T_) Values for Baseline [^18^F]GEH200449 PET Measurements (*n* = 4) Obtained by the Two-Tissue Compartment (2-TC) Model and the Logan Graphical Analysis^a^

	*V*_T_, 2-TC		*V*_T_, Logan	
brain region	mean	range	mean	range
caudate nucleus	22.0	14.6–34.1	18.9	15.0–22.9
putamen	17.3	14.1–21.1	16.5	14.6–18.9
thalamus	19.8	15.0–27.7	17.3	14.3–20.0
occipital cortex	11.0^*^	10.8–11.2*	11.0	10.3–12.1
prefrontal cortex	14.7	11.0–20.4	13.1	11.5–15.4
cerebellum	13.6^**^	11.1–16.0^**^	12.6	11.2–13.9
whole brain	13.5	10.9–17.3	12.4	11.2–14.1

a 2-TC model yielded implausible estimates of *V*_T_ (>500 mL cm^–3^; SE > 1000%) for occipital cortex in NHPs #4 and #5 (*; *n* = 2) and for the cerebellum in NHP #4 (**; *n* = 3).

Based on visual inspection of the PET images (Figure 2) and comparison of regional *V*_T_ values at baseline and post-drug administration (Figure S4), inhibition of radioligand binding was observed for all brain structures, indicating the absence of suitable reference region for quantification of radioligand binding. The occupancy at MAO-B was calculated for the pretreatment experiments with *L*-deprenyl and rasagiline. Based on a graphical analysis of *V*_T_,^22^ the occupancy of *L*-deprenyl was 97 and 82%, respectively, at the 0.5 and 1.0 mg/kg dose levels. The reason for the lower occupancy estimated at the 1.0 mg/kg dose level than at 0.5 mg/kg of *L*-deprenyl is not known. However, given that the occupancy calculations were based on studies in two different NHPs, it is possible that the discrepancies may partly reflect intersubject variability in drug plasma exposure. In addition, possible effects of *L*-deprenyl on plasma protein binding of [^18^F]GEH200449 cannot be excluded. The corresponding value for rasagiline at the 0.75 mg/kg dose level was 97% (Figure S4). Occupancy could not be calculated for 0.25 mg/kg rasagiline, since arterial plasma samples were not available for this measurement.

## Conclusions

The novel MAO-B radioligand [^18^F]GEH200449 was evaluated using PET in NHPs. The brain exposure of [^18^F]GEH200449 was high and the regional brain distribution was consistent with the known localization of MAO-B. The binding of [^18^F]GEH200449 could be inhibited by the administration of reference MAO-B ligands before or after radioligand administration. The Logan graphical analysis was selected as the preferred method for the quantification of [^18^F]GEH200449 binding. These observations support that [^18^F]GEH200449 is a reversible MAO-B radioligand suitable for applied studies in humans.

## Material and Methods

### Radiochemistry

The precursor (GEH200452) and the non-radioactive reference standard (GEH200449) were supplied by GE Healthcare. [^18^F]GEH200449 was synthesized from the precursor GEH200452 as previously described.^18^ The radiochemical purity of [^18^F]GEH200449 was >99% at the time of administration and the molar radioactivity was in the range of 12–134 GBq/μmol corresponding to an injected mass of 0.3–3.5 μg (Table S1).

### Non-Human Primates

This study was approved by the Animal Ethics Committee of the Swedish Animal Welfare Agency (Dnr 145/08, 399/08, and 386/09) and was performed according to the “Guidelines for Planning, Conducting and Documenting Experimental Research” (Dnr 4820/06-600) of the Karolinska Institutet and the “Guide for the Care and Use of Laboratory Animals”.^23^

Two male and three female NHPs (#1–5) of the species *M. fascicularis*, weighing 4.2–8.8 kg, were supplied by the Astrid Fagraeus Laboratory, Karolinska Institutet, Solna, Sweden. Anesthesia was initiated by intramuscular injection of ketamine hydrochloride (ca. 10 mg/kg, Ketalar, Pfizer) and maintained by intravenous infusion of a mixture of ketamine hydrochloride (4 mg/kg/h) and xylazine hydrochloride (0.4 mg/kg/h Rompun Vet., Bayer).

Heart and respiration rates were continuously monitored and body temperature was maintained by a Bair Hugger heater—Model 505 (Arizant Healthcare Inc., MN) and monitored with an esophageal thermometer. At anesthesia, the head was immobilized in a head fixation system^24^ and the NHP was positioned in the gantry of the PET system.

### PET Data Acquisition

PET measurements were conducted using the high-resolution research tomograph system (Siemens Molecular Imaging, Knoxville, TN, USA) for NHPs #1–4 and using the LFER 150 PET/CT system (Mediso Ltd., Budapest, Hungary) for NHP #5.

Each PET measurement was performed on a separate experimental day. Experiments conducted in the same NHP were separated by at least 6 weeks. Baseline PET measurements were initially undertaken in each of the five NHPs. Drug inhibition binding studies were then carried out using the two selective MAO-B inhibitors, *L*-deprenyl^25^ and rasagiline,^26^ and the two mGluR5 compounds, fenobam and AZD9272, that recently have been found to have high affinity toward MAO-B.^21^ PET measurements were conducted in two NHPs after administration of *L*-deprenyl (NHP #1, 1.0 mg/kg; NHP #4, 0.5 mg/kg), in one NHP after administration of rasagiline (NHP #4, 0.25 and 0.75 mg/kg), in one NHP (#5) after administration of 1.0 mg/kg of fenobam and in one NHP (#3) after the administration of 0.15 mg/kg AZD9272 (Table S1). Test compounds were administered as a 10 min intravenous infusion starting 45 min prior to the PET measurement for *L*-deprenyl and rasagiline and 15 min prior to the PET measurement for fenobam and AZD9272 based on earlier investigations.^21,27^

In addition, to confirm the reversibility of [^18^F]GEH200449 binding displacement PET measurements using 0.5 mg/kg of *L*-deprenyl administered as a 10 min intravenous infusion 25 min after radioligand injection were undertaken in two NHPs (#2 and #4). Further experimental details are provided in Table S1.

At start of PET data acquisition, a sterile physiological phosphate buffer solution (pH = 7.4) of [^18^F]GEH200449 (injected radioactivity, 107–167 MBq) was injected as a bolus into a sural vein. Emission data were acquired in list mode for 123 min. Arterial blood was sampled as previously described^28^ using an automated blood sampling system (ABSS) during the first 3 min of each PET measurement. Subsequently, arterial blood samples (0.7–2.5 mL) were manually drawn at 7, 15, 30, 60, 90, and 120 min after injection of [^18^F]GEH200449. After centrifugation, 0.15–1.0 mL of plasma was pipetted and plasma radioactivity was measured in a well counter. In addition, samples were taken directly from the ABSS at 0.5, 1, 1.5, 2, 2.5, and 3 min for cross-calibration with the well counter and for the determination of the plasma-to-blood ratio.

The fraction of plasma radioactivity corresponding to the unchanged radioligand in plasma was determined from arterial plasma samples collected at 2, 7, 15, 30, 60, 90, and 120 min after injection of [^18^F]GEH200449 according to previously described procedures.^29^

### PET Data Analysis

Dynamic images were reconstructed as previously described.^30,31^ Regions of interest (ROIs) for the whole brain and selected regions (caudate nucleus, putamen, thalamus, occipital cortex, prefrontal cortex, and cerebellum) were manually delineated on T1-weighted magnetic resonance images (MRIs) acquired as previously described.^21,27^ PET images were coregistered to the MRIs, and time-activity curves were generated by pooling ROIs for each paired anatomical region and applying the pooled ROIs to the coregistered PET images. Delineation of ROIs and image coregistrations were performed using the software PMOD v. 3.6 (PMOD Technologies, Zurich, Switzerland).

Time curves for regional [^18^F]GEH200449 binding at baseline were analyzed by kinetic modeling using 1-TC and 2-TC models,^32^ and *V*_T_ as outcome measures. Akaike information criterion^33^ and F statistics were applied to select the preferred model. Regional estimates of *V*_T_ for [^18^F]GEH200449 were also obtained using the Logan linear graphical method^34^ with t* fixed at 40 min. The analyses were performed using the kinetic modeling tool in PMOD v. 4.3. Occupancy at [^18^F]GEH200449 binding sites was estimated based on regional *V*_T_ values obtained by the Logan method at baseline and after drug administration according to a graphical procedure described in the literature.^22^

## Data Availability

The data will be made available upon request.

## Supporting Information Available

Experimental details of PET studies, statistics comparing 1-TC and 2-TC kinetic models and 2-TC parameter estimates for [^18^F]GEH200449 data, time-curves of whole brain radioactivity concentration for [^18^F]GEH200449 at baseline, time-curves for regional brain radioactivity of [^18^F]GEH200449 at baseline and after administration of AZD9272 or fenobam, time-curves for percentage of parent (unmetabolized) radioligand and chromatograms of radiometabolism at 2 and 15 min after radioligand injection, result of graphical analysis of drug-induced occupancy for *L*-deprenyl or rasagiline (PDF)

## Abbreviations

1-TC: one-tissue compartment
2-TC: two-tissue compartment
ABSS: automated blood sampling system
MAO-B: monoamine oxidase B
mGluR5: metabotropic glutamate receptor 5
NHPs: non-human primates
PET: positron emission tomography
ROIs: regions of interest
SUV: standardized uptake value
*V*_T_: total distribution volume
