# Supplementary material for: PET Evaluation of the Novel F-18 Labeled Reversible Radioligand [18F]GEH200449 for Detection of Monoamine Oxidase-B in the Non-Human Primate Brain
Source: ACS Chem Neurosci. 2023 Aug 17;14(17):3206–11. doi: 10.1021/acschemneuro.3c00332 (PMC10485887; doi:10.1021/acschemneuro.3c00332)
Supplement: Supplementary file 1 — cn3c00332_si_001.pdf [file cn3c00332_si_001.pdf]

PET Evaluation of the Novel F-18 Labeled Reversible Radioligand [^{18}F]GEH200449 for Detection of Monoamine Oxidase-B in the Non-Human Primate Brain

Katarina Varnäs, Sangram Nag, Christer Halldin, Lars Farde

Karolinska Institutet, Department of Clinical Neuroscience, Center for Psychiatry Research and Stockholm County Council, BioClinicum J:15, Visionsgatan 4, SE-171 64 Solna, Sweden

Supporting Information

Supplementary Tables

Table S1. Description of PET studies conducted using the radioligand [^{18}F]GEH200449 in non-human primates (NHP).

NHP #	Experimental condition	Body weight (kg)	ID (MBq)	MA (GBq/µmol)	Injected mass (µg)	Plasma sampling
1	Baseline	6.6	159	46	0.9	Arterial
	Pretreatment *L*-deprenyl, 1.0 mg/kg	6.8	138	36	1.0	Arterial
2	Baseline	8.8	107	74	0.4	Venous
	Displacement *L*-deprenyl, 0.5 mg/kg	8.6	167	58	0.8	Venous
3	Baseline	6.3	165	65	0.7	Arterial
	Pretreatment AZD9272, 0.15 mg/kg	5.9	161	99	0.4	Venous
4	Baseline	4.6	149	66	0.6	Arterial
	Pretreatment *L*-deprenyl, 0.5 mg/kg	4.7	156	69	0.6	Arterial
	Displacement *L*-deprenyl, 0.5 mg/kg	4.2	147	134	0.3	Arterial
	Pretreatment rasagiline, 0.25 mg/kg	4.7	160	12	3.5	Venous
	Pretreatment rasagiline, 0.75 mg/kg	4.9	167	60	0.8	Arterial
5	Baseline	5.1	116	35	0.9	Arterial
	Pretreatment fenobam, 1.0 mg/kg	5.2	137	29	1.3	Venous

ID, Injected radioactivity; MA, molar activity.

Table S2. Statistics comparing kinetic 1- (1-TC) and 2-tissue compartment (2-TC) models and 2-TC parameter estimates for [^{18}F]GEH200449 data.

NHP #	Brain region	Preferred model based on lower AIC	F statistic (p-value) 2-TC vs. 1-TC	2-TC parameter estimates K_1 (mL min^{-1} cm^{-3})	k_2 (min^{-1})	k_3 (min^{-1})	k_4 (min^{-1})	V_T (mL cm^{-3})
1	CAU	2-TC	30.55 (< 0.0001)	0.70	0.09	0.034	0.037	14.6
	CER	2-TC	28.25 (< 0.0001)	0.47	0.07	0.021	0.038	11.1
	OC	2-TC	68.40 (< 0.0001)	0.49	0.07	0.016	0.024	10.8
	PFC	2-TC	23.57 (< 0.0001)	0.57	0.09	0.028	0.037	11.0
	PUT	2-TC	22.83 (< 0.0001)	0.71	0.09	0.025	0.029	14.1
	THA	2-TC	20.53 (< 0.0001)	0.59	0.06	0.008	0.016	15.0
	WB	2-TC	39.28 (< 0.0001)	0.51	0.09	0.025	0.031	10.9
3	CAU	2-TC	8.24 (0.0014)	0.65	0.04	0.006	0.029	19.2
	CER	2-TC	12.34 (0.0001)	0.44	0.04	0.005	0.016	13.9
	OC	2-TC	7.90 (0.0018)	0.38	0.04	0.003	0.012	11.2
	PFC	2-TC	65.27 (< 0.0001)	0.49	0.05	0.006	0.017	13.5
	PUT	2-TC	24.68 (< 0.0001)	0.70	0.05	0.007	0.029	16.7
	THA	2-TC	29.69 (< 0.0001)	0.58	0.04	0.007	0.023	18.5
	WB	2-TC	44.76 (< 0.0001)	0.48	0.05	0.010	0.029	12.6
4	CAU	1-TC	0.43 (0.6543)	0.50	0.05	1.087	1.229	19.9
	CER	1-TC	2.34 (0.1134)	0.32	0.03	0.001	0.000	NA
	OC	2-TC	15.22 (< 0.0001)	0.31	0.03	0.001	0.000	NA
	PFC	2-TC	11.24 (0.0002)	0.37	0.03	0.002	0.013	13.9
	PUT	2-TC	5.92 (0.0068)	0.57	0.04	0.006	0.034	17.2
	THA	2-TC	14.48 (< 0.0001)	0.53	1.24	4.043	0.098	18.1
	WB	2-TC	53.39 (< 0.0001)	0.34	0.03	0.003	0.016	13.1
5	CAU	2-TC	32.85 (< 0.0001)	0.93	0.05	0.004	0.004	34.1
	CER	2-TC	41.95 (< 0.0001)	0.51	0.05	0.006	0.011	16.0
	OC	2-TC	8.22 (0.0014)	0.37	0.04	0.002	0.000	NA
	PFC	2-TC	43.50 (< 0.0001)	0.61	0.05	0.004	0.006	20.4
	PUT	2-TC	36.62 (< 0.0001)	0.86	0.06	0.007	0.012	21.1
	THA	2-TC	57.03 (< 0.0001)	0.64	0.04	0.004	0.005	27.7
	WB	2-TC	314.46 (< 0.0001)	0.50	0.05	0.005	0.008	17.3

NHP, non-human primate; CAU, Caudate nucleus; CER, cerebellum; OC, occipital cortex; PFC, prefrontal cortex; PUT, putamen; THA, thalamus; WB, whole brain; NA, not applicable *(V_T >500 mL cm^{-3})*.

Supplementary Figures

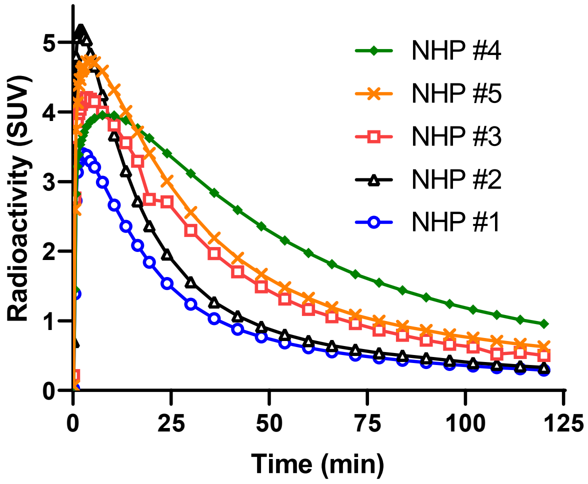

Figure S1. Whole brain radioactivity concentration versus time for [^{18}F]GEH200449 at baseline.

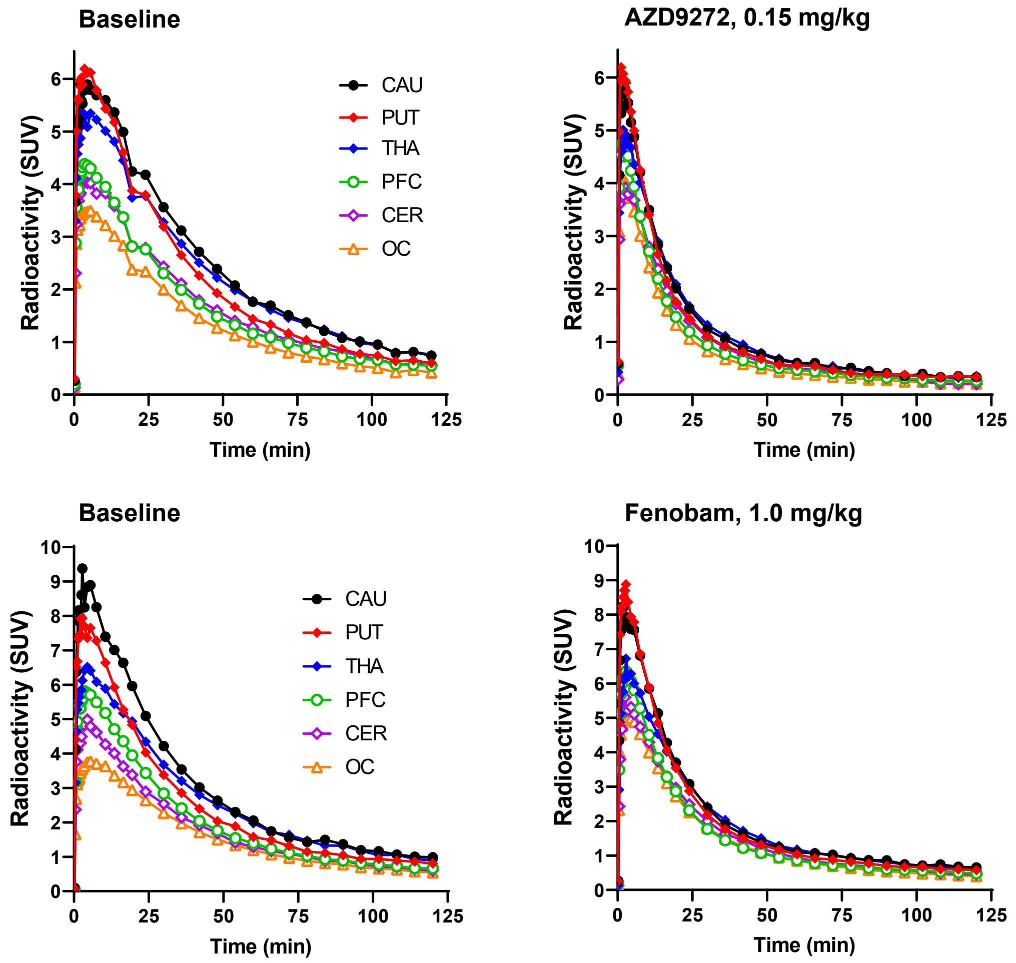

Figure S2. Regional brain radioactivity after i.v. injection of [^{18}F]GEH200449 at baseline and after administration of AZD9272, 0.15 mg/kg in NHP #3 (upper panel), or after administration of fenobam, 1.0 mg/kg in NHP #5 (lower panel). CAU, Caudate nucleus; PUT, putamen; THA, thalamus; PFC, prefrontal cortex; CER, cerebellum; OC, occipital cortex.

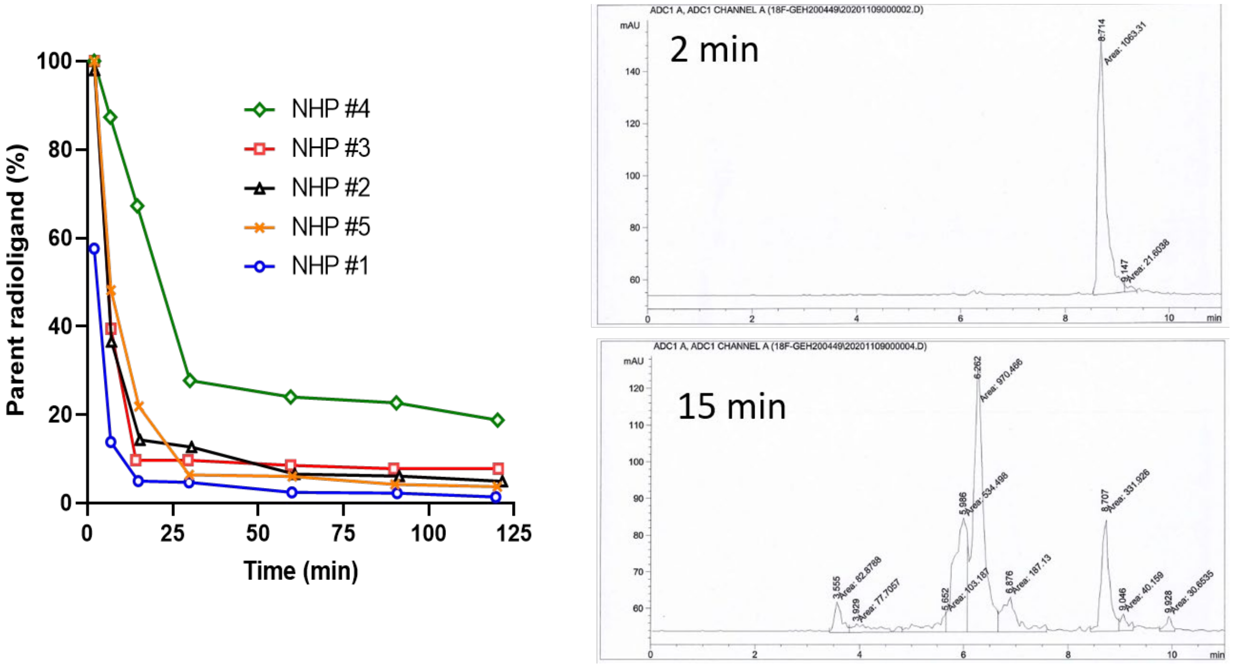

Figure S3. Radiometabolite analysis of [^{18}F]GEH200449 in NHP plasma. Left, percentage of total radioactivity of parent radioligand versus time at baseline. Data for arterial samples are shown for NHPs #1, #3, #4 and #5, and data for venous samples are shown for NHP #2. Right, radiochromatogram of radiometabolism at 2 and 15 minutes after radioligand injection in NHP #2, baseline measurement.

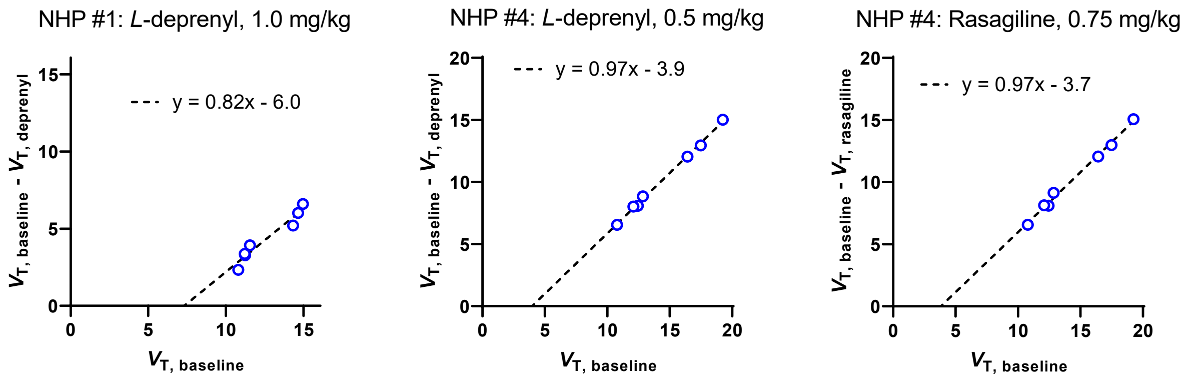

Figure S4. PET studies of drug-induced occupancy of *L*-deprenyl or rasagiline at [^{18}F]GEH200449 binding sites in the non-human primate brain. Occupancy was estimated from the slope of a graphical analysis (Cunningham et al., 2010).

References

Cunningham et al., 2010. Measuring drug occupancy in the absence of a reference region: the Lassen plot re-visited. J Cereb Blood Flow Metab 30: 46-50.
